# Salinity-driven niche partitioning of aquatic viruses in one of Europe’s largest estuaries

**DOI:** 10.1128/aem.00807-26

**Published:** 2026-06-10

**Authors:** Julia Gołębiowska, Jason N. Woodhouse, Sven P. Tobias-Hünefeldt, Hans-Peter Grossart

**Affiliations:** 1 Department of Plankton and Microbial Ecology, Leibniz Institute of Freshwater Ecology and Inland Fisheries (IGB), Stechlin, Germany; 2 Department of Microbiology and Biotechnology, Institute for Plant Sciences and Microbiology, University of Hamburg 14915, Hamburg, Germany; 3 Department of Molecular Animal Physiology, Institute for Animal Cell and Systems Biology, Hamburg, Germany; 4 Institute of Biochemistry and Biology, Potsdam University, Potsdam, Germany; University of Delaware, Lewes, Delaware, USA

**Keywords:** salinity gradient, Elbe Estuary, carbon cycle, metagenomes, metatranscriptomes, viral infection, aquatic viruses, bacteriophages, megaviruses, satellite viruses

## Abstract

Viruses are a vital part of the aquatic food web and hold a profound role in carbon and energy cycling at different trophic levels. Despite the rising interest in aquatic viruses, very few studies were conducted in estuaries, where freshwater and marine communities meet along the salinity gradient. We present a paired analysis of metagenomic and metatranscriptomic data focusing on the viral fraction derived from seasonal sampling between May 2021 and November 2022 in one of Europe’s largest estuaries, the temperate mesotidal Elbe River downstream of Hamburg. Our results reveal a sharp delineation of viral communities along specific salinity niches and provide evidence for their adaptation. This implicates viruses as a structural component of microbial and phytoplankton ecology across the estuary. We provide a detailed overview of the spatiotemporal distribution of viruses, including taxonomy and hosts, which emphasizes the role of giant viruses (Megaviricetes) in waters of lower salinity and RNA viruses in marine environments. We identify, besides salinity, total dissolved phosphate and temperature as the main drivers of estuarine viral communities. We find a broad spectrum of metabolic pathways, potentially altered by viruses via auxiliary metabolic genes. Potential metabolisms impacted included the underlying carbon processes like photosynthesis or methane metabolism, but may also extend to some xenobiotics and antibiotics metabolisms in this anthropogenically altered estuary. This is the first detailed molecular study of viruses in the Elbe Estuary, shedding light on viral communities and their ecological roles in controlling microbial populations at the base of the estuarine food web.

**IMPORTANCE:**

Estuaries are the interfaces between marine and limnic waters, with their own specific hydrological and biochemical processes due to, e.g., salinity gradients, tides, and terrestrial inflows. In particular, they are sites of intensive carbon cycling. Their often high economic importance causes substantial anthropogenic pressure on the ecosystem. All of these result in extremely complex factors interacting and influencing microbial populations. Our study provides a first comprehensive overview of the viral communities in Europe’s largest estuary. We made an attempt to disentangle the numerous environmental parameters, and we highlight salinity as the most important factor, providing evidence of its multidimensional influence on the estuarine virome. Our findings deepen our understanding of viral communities and their interactions with microbes and bring us a step closer to their role in aquatic food webs, particularly in carbon turnover in estuaries.

## INTRODUCTION

Aquatic viruses are known for their multidimensional effects on living organisms. Viral infections affect the natural ecosystem and economic viability of aquaculture (1). Viruses exhibit a strong top-down effect on microbial biodiversity, either through “kill-the-winner” strategies, which eliminate the most dominant members of the population and create new niche opportunities for less abundant individuals, or by becoming agents of horizontal gene transfer, enriching the genetic pool of invaded organisms (2). Viruses can also regulate microbial diversity through bottom-up effects via the “viral shunt.” Viral lysis of microbial organisms transforms particulate organic matter (POM) into dissolved organic matter. This release of fresh organic matter increases its circulation through the microbial loop boosting both phytoplankton and bacterioplankton production. However, it can also lead to carbon burial in the deep ocean (3, 4) by accelerating the biological carbon pump (5). Viruses can further impact export of POM either by increasing aggregation due to the release of adhesive organic polymers or by facilitating particle dispersion and resuspension in the water column (2, 6, 7). Therefore, particle-associated viruses potentially function as aggregate disruptors, while those in the surrounding water could function as aggregators. Different clonality and host densities between free-living and particulate fractions could develop different infection strategies (8) and lead to viral speciation. These aspects have been widely explored in marine and freshwater ecosystems (2, 6, 8–11), but very few studies have been conducted in estuaries (12, 13) where freshwater and marine waters intermix.

Estuaries are ideal study systems exhibiting a great degree of environmental heterogeneity with both short- and long-term dynamics driven by changes in the relative contribution of freshwater flows, marine tidal influence, and terrestrial runoff. They are also relevant sites of dynamic carbon cycling. On the one hand, they are part of the blue carbon burial sites, which are vegetated coastal zones sequestering carbon (14). On the other hand, they are responsible for a substantial fraction of the global CO_2_ production via high respiration rates (15–17). The dynamic hydrological changes due to currents, tides, and seawater intrusion in the estuaries can be used to observe effects of extreme events that are increasing in frequency with global change. The varying salinity levels along the estuarine continuum can be useful to understand consequences of salinization accelerated by global warming and urbanization (18). Furthermore, they are suitable for studying the influence of salinity on ecoevolutionary dynamics, greatly overlooked at the viral population level. For viruses, it is known that salinity affects their infectibility and lytic-lysogenic equilibrium (19–22), but more detailed effects on their composition along salinity gradients are missing.

The Elbe Estuary is representative of a typical mesotidal temperate estuary, including a highly diverse morphology. The estuary connects the Elbe River, via Hamburg Harbor, with the North Sea and has historically represented a site of economic and cultural importance. As a consequence, the Elbe Estuary is one of the world’s most monitored but also most manipulated estuarine ecosystems, with extensive background data characterizing flow rates, rainfall, water quality, and phytoplankton data. In recent years, the RTG2530 graduate school has been instrumental in expanding our knowledge of the Elbe Estuary to better understand the role of physical and biological processes in estuarine carbon cycling. These studies include, but are not limited to, the influence of salinity gradients on microbial carbon processes in the water and marshland, phytoplankton diversity, and fish population dynamics and fitness (23–27).

Despite extensive efforts to understand the role of different trophic levels on estuarine carbon cycling, viruses still remain largely underexplored. The current state of knowledge of coastal viruses points to estuaries as sites of increased viral production and infection rates compared to rivers and open oceans (28). These studies suggest an important role of local-estuarine hydrological and climatic factors, e.g., winds, heavy rains, tides, stratification, and mixing of riverine and sea water for viral persistence and progeny. Globally, estuaries exhibit differences in viral abundance and productivity, depending on latitude (29). Viral production increases within the estuary with salinity (29–31) and varies on different temporal scales, e.g., with seasonality (29, 31–33) and tides (30). These factors, by affecting viral production and lytic infection rate, can substantially influence carbon dynamics in the estuaries (30). Potentially, they are also sites of novel viruses (34, 35).

This study investigates the viral communities along the salinity gradient of the Elbe Estuary. Previous studies identified strong mesotidal influences on microbial community composition and functions (36), while temporal differences dominated carbon dynamics (26). We are presenting one of the very first detailed overviews of estuarine viruses by means of metaomics (35, 37). Compared to previous studies, we integrated metagenomics with metatranscriptomic data to capture RNA viruses and the activity of DNA viruses. In particular, we addressed the following questions. (i) How does salinity shape viral communities on the genetic level? (ii) Which viral groups and associated processes are characteristic of different ecological niches along the Elbe Estuary?

## MATERIALS AND METHODS

### Sampling and data acquisition

**Fig 1:**
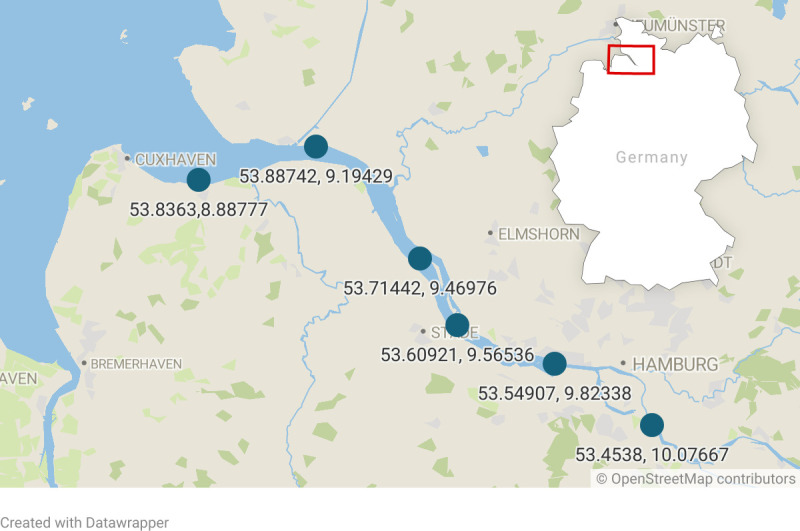
Map of the mesotidal Elbe Estuary with marked sampling stations.

For a full overview of sampling procedures and DNA extraction and sequencing, see the relevant section in Materials and Methods in Tobias-Hünefeldt et al. (36). In brief, samples were taken from the River Elbe Estuary from May 2021 to November 2022 at five to six consecutive stations (Fig. 1) with a horizontal sampler at a depth of 1 m. Suspended and sinking particles were separated via sedimentation for 30 minutes, and downstream particle analyses were carried out on each separate fraction. Free-living microbes were captured on 0.22 µm Durapore filters, using the filtrate from particle-associated microbes captured on 5.0 µm Durapore filters. All samples were collected in duplicate, unless otherwise stated.

DNA was extracted using the phenol-chloroform method described in Nercessian et al. (38). For cell lysis, the CTAB buffer, sodium dodecyl sulfate, and N-Lauroylsarcosine (anion surfactants), and proteinase K were added. Final cell disruption was aided by zirconia beads and FastPrep instrument. Polyethylene glycol was used for DNA purification and precipitation at 4°C. Finally, the pellet was washed in ethanol and dissolved in Tris. Samples were prepared for sequencing with the Illumina DNA Prep Kit and sequenced on a NovaSeq 6000 platform (Illumina, San Diego, CA, USA). One hundred ninety metagenomic samples and 73 metatranscriptomic samples were obtained, and 13,765 prokaryotic metagenome-assembled genomes (MAGs) were recovered. A detailed description of the process used to generate MAGs and non-redundant gene catalogs can be found in Tobias-Hünefeldt et al. (36).

### Virome detection and annotation

In addition to the MAG-derived gene catalog, we sought to amend this data set with viral-derived genes sourced directly from both metagenome and metatranscriptome assemblies. To this end, we used VIBRANT (v1.2.0) (39) integrated into the ViWrap pipeline (v1.3.0) (40) with default parameters to extract the virome from shotgun sequencing data of the abovementioned microbial-size fractions, assess genome completeness, annotate viral genes based on Kyoto Encyclopedia of Genes and Genomes (KEGG, v2019-03-20) (41), Pfam (v32) (42), and Viral Orthologous Group (VOG) (release 94) (43) databases, with a minimum scaffold length of 2,000 bp, and perform auxiliary genes detection with their assignment to KEGG metabolic pathways. All viral genes annotated by VIBRANT were subsequently clustered at 95% identity, keeping the longest sequence as representative using CD-HIT (v4.8.1) with the parameters -c 0.95 -M 0 -G 0 -aS 0.9 -g 1 -r 0 -d 0 -b 1000. This non-redundant viral gene catalog was merged with the MAG gene catalog using CD-HIT (v4.8.1) using the cd-hit-est-2d function and a 95% ANI. Pre-processed, quality controlled metagenome and metatranscriptome reads were mapped back against this gene catalog and normalized using prokaryotic single-copy marker genes (44). The resulting abundances of viral genes/transcripts are then expressed as a copy number per prokaryotic genome/cell. For the visualization (Fig. 6), we selected the most expressed genes, which cumulatively constituted 80% of total expression calculated by the sum of logarithms of transcriptomic counts divided by gene counts. We used the CheckV (v1.0.1) (45) “end_to_end” program with default parameters for the quality assessment of detected viral contigs based on completeness according to MIUViG (46) quality categories and cutoffs, i.e., “complete,” “high quality” (>90% completeness), “medium quality” (50%–90% completeness), “low quality” (<50% completeness), and “undetermined quality.” We removed viral contigs flagged as “low” or “undetermined” quality, as well as contigs shorter than 10 kbp. The remaining viral contigs were grouped into 2,372 viral operational taxonomic units (vOTUs) (95% ANI) using dRep (47). Due to the high variability of eukaryotes and prokaryotes in our samples, we sought to express the abundance of vOTUs also as a function of bacterial genome copy number rather than as a function of sampling depth (RPKM/TPKM). Considering the differences in completeness and length of viral contigs grouped into one vOTU, we opted to identify vOTU specific marker genes for quantification, rather than map reads against a single “representative” viral contig for vOTUs. We were able to identify a single unique marker gene that was specific for a single viral genome cluster for 99.54% of clusters and 99.75% of viral contigs. Because of the challenges in the virus quantification, especially proviruses, which are usually under detection level (48), the VOGs were favored over others. Finally, 99.66% of all marker genes belonged to the Database of Virus Orthologous Groups (49). Per prokaryotic genome, abundances of cluster-specific viral marker genes were extracted from our previously quantified gene catalog and used to represent changes in viral community composition. Calculation of Shannon, Simpson, and observed OTU indexes of alpha diversity based on the unique marker gene counts was performed using the phyloseq R package (50). The counts were first transformed by multiplying by 100 so that the observed OTU alpha-diversity index could be calculated. The Kruskal–Wallis test and post hoc Dunn test were applied to assess differences between groups.

### Taxonomy, niche assignment, and genome length estimation

Viral taxonomy of the contigs detected by VIBRANT was assigned by the geNomad software (v1.8.1) and unified at a level that accounted for more than 50% of all sequences per vOTU (51). As it was reported that geNomad is better suited for giant virus (Nucleocytoviricota) detection (52), we compared the percentage of contigs assigned as giant viruses between the set of contigs detected by VIBRANT (see “Virome detection and annotation,” above) and the set of contigs detected by geNomad but not by VIBRANT. The results were 2.97% and 2.39%, respectively, meaning that giant viruses were not enriched in the newly analyzed data set. Almost all (96.87%) of the contigs detected by VIBRANT and annotated by geNomad overlapped with the set detected by geNomad, indicative of a low false discovery rate in the presented taxonomy overview. To further improve the taxonomy annotation of giant viruses, we used the geNomad annotated giant viruses marker genes according to Aylward et al. (53), which we used as a query against the GVDB (53) using the mmseqs2 easy search program (54). We filtered out the results above 10^−10^ e-value, and we took into account the 10 best hits based on e-value and identity. This enabled us to more precisely annotate 8 out of 16 giant viruses’ vOTUs.

Genome sizes were estimated by dividing a contig’s length by its completeness according to CheckV. For niche assignment, salinity levels were set according to the common definition and k-medoid clustering of PSU values, i.e., freshwater, 0–0.5 PSU; oligohaline, 0.5–5.5 PSU; mesohaline, 5.5–18 PSU; and polyhaline, 18–30 PSU. Assembled genome salinity level was assigned according to original sample metadata, with a general niche assignment per primary cluster at a cutoff of 80%. If this cutoff was not satisfactory, 20% cutoffs were used to assign a broader niche. The same procedure was used to find particle niches. Venn diagrams required clusters of more than four genomes.

### Host prediction

Hosts of contigs annotated as phages were assigned by Integrated Phage-Host Prediction (iPHoP v1.3.3) (55), with a score cutoff of ≥90 and a database completed with prokaryotic MAGs assembled from the original Elbe Estuary metagenomic data, with assignments performed at the genus and genome levels (Fig. S1 and S2). The generalist vs specialist analysis was based on the output with a resolution to host species. All assigned phyla per cluster were used to obtain host range and distribution of viruses according to their hosts. Hosts of eukaryotic viruses were assigned by running the MMseqs2 easy-search workflow, with the parameters --search-type 3 --start-sens 2 --sens-steps 3 -s 7 (54) against Virus-Host DB (52), with manually curated hosts based on the literature. The results were further filtered by an e-value of <0.0001.

### Different viromic data sets and their dimensionality reduction

In this study, different data sets are used, as they represent different aspects of the viral community and ecology. Namely, the subsetted genes/transcripts assigned as viral resulted in viral metagenomic data representing the viral metabolic potential of DNA viruses, paired metatranscriptomic data representing the virus transcriptomic activity and metabolic potential of RNA viruses, and normalized transcriptomic data resulting in viral expression. From the mentioned data sets, the unique marker genes of the viral cluster were subsetted, and respective data sets were obtained: vOTUs representing DNA virus diversity, vOTU transcripts—activity of viral community and viral diversity of RNA viruses, and vOTU expression—activity of DNA viruses normalized using metagenome copy numbers.

PCA was performed by “PCA” from the FactoMineR R package on log-transformed viral metagenomes, metatranscriptomes, and expression with an added pseudocount equal to 0.000001. Analysis of similarities (ANOSIM) tests using the “anosim” function from vegan package with 9,999 permutations were based on the first two PC values. Principal coordinate analysis (PCoA) was performed on the phyloseq object of vOTU tables using the “plot_ordination” function from the same package. To perform the ANOSIM test, Bray–Curtis dissimilarity matrices were obtained from vOTU data sets, applying the phyloseq “distance” function.

Canonical correspondence analysis was performed by the “cca” function from the vegan package (2.6-6) (56) on unique marker genes. The lacking parameter values were imputed based on linear models, including selected present parameters. Environmental factors were selected based on empirical *P* values (≥0.001) calculated by “envfit” from the same package with 999 permutations. Figures were generated with the ggplot2 package (v3.5.2) (57), unless otherwise stated.

Mantel tests from the vegan package assessed the correlation between environmental parameters, OTU, and OTU expression of viral, microbial, and host communities within different fractions with 9999 permutations. Host communities were obtained by subsetting microbial communities based on MAGs assigned as hosts by iPHoP. To obtain pairwise distance matrices of subsampled MOTUs (microbial communities and their subset—host communities), the “avgdist” function was applied based on Bray–Curtis dissimilarity, whereby subsampling depth equals 500 and iterations equal 100. In the other cases, the “vegdist” function was applied based on Bray–Curtis dissimilarity. *P* values were Hochberg adjusted unless otherwise stated.

### Major capsid protein trees

The phylogenetic tree was based on the Clustal Omega (v1.2.4) (58) alignment of amino acid sequences annotated as major capsid proteins (MCPs) by VIBRANT. The tree was calculated by IQ-TREE (v2.4.0) (59) with 1,000 ultra-fast bootstraps. VT + R10 was chosen as the best model according to the Bayesian information criterion with a model finder option. The obtained model was used for recalculating separate trees of different MCPs. The resulting MCP trees were analyzed with ape (5.8-1) (60) and visualized by ggtree (3.10.1) (61) R packages.

### Isoelectric point distribution

The isoelectric points of viral proteome and MCPs were calculated by “computePI” from the seqinr (62) R package on sequences detected by VIBRANT.

## RESULTS

### Characterization of viral genomes

We detected 963,440 viral contigs in all microbial-size fractions (free-living, –0.22 to 5 µm, and particle-associated suspended and sinking fractions, >5 µm), among which 7,714 represent medium-quality genomes, 2,759 represent high-quality genomes, and 1,219 represent complete genomes. Finally, we obtained 2,372 vOTUs. We assigned various hosts to 655 vOTUs annotated as phages, 533 of which were assigned to only one host phylum and 407 to only one host lineage (Fig. S1 and S2). Among 23 eukaryotic vOTUs with assigned hosts, 9 had been assigned to a single host lineage (Fig. S3). We detected 1,905,363 viral gene clusters and among those, 14,601 were assigned as auxiliary genes and were assigned to distinct metabolic pathways.

### Impact of salinity on viral taxonomy diversity and function along the estuarine gradient

**Fig 2:**
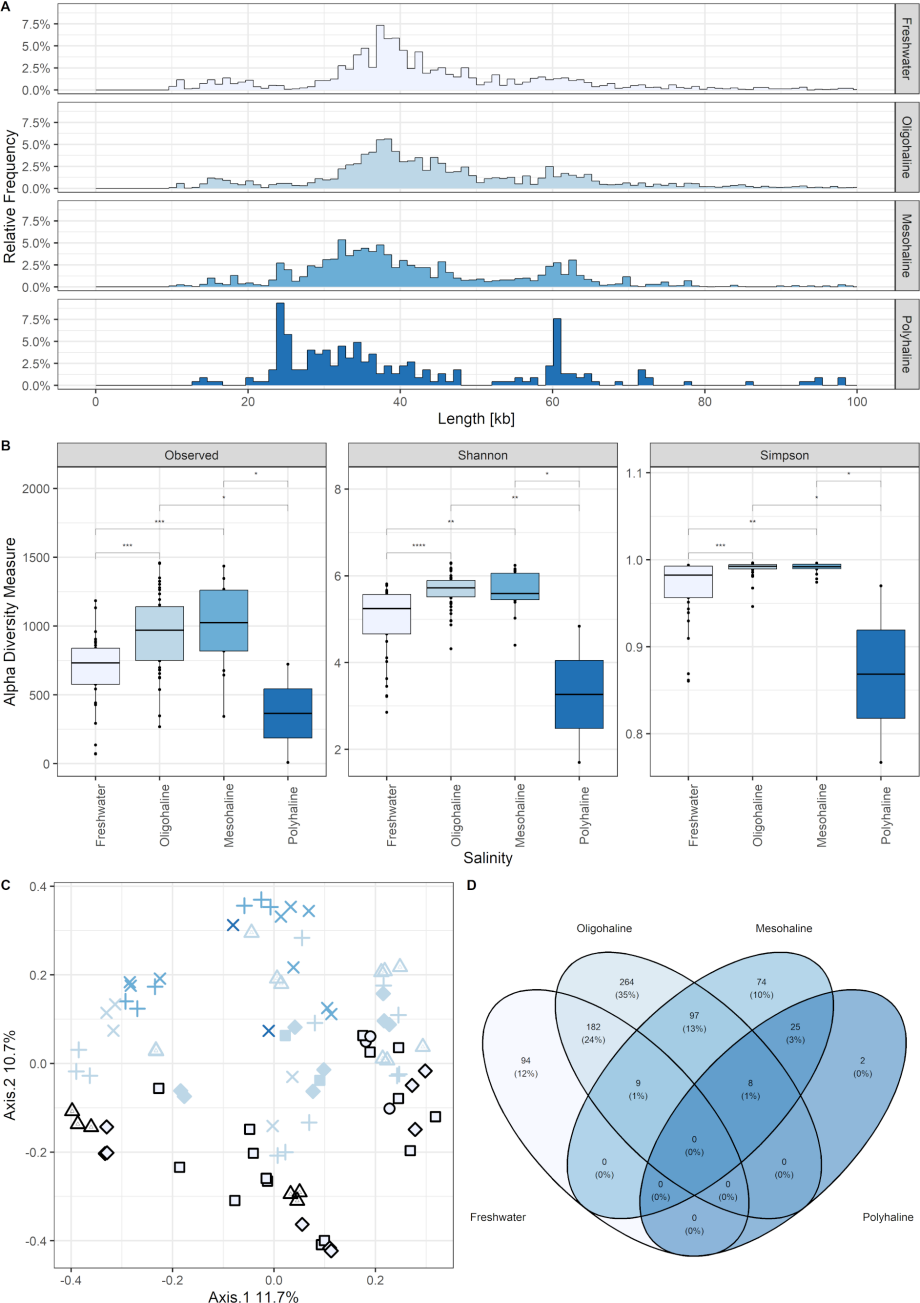
vOTU diversity. (**A**) Estimated viral genome length distribution by salinity levels. (**B**) Comparison of alpha-diversity indexes between different salinity levels; stars denote *P* values from Dunn’s test ( *P < 0.05, **P < 0.01, ***P <0.001, ****P < 0.0001). (**C**) Bray–Curtis distance-based PCoA representing beta diversity. shapes represent different sampling stations. (**D**) Venn diagram representing the number and percentages of viral clusters belonging to different salinity niches.

The estimated viral genome length varied from 10,031 to 457,441 bp, and this range was shared between different salinity levels. All salinity levels displayed peaks at 35–37 kb and 60 kb, although the presence of a 25 kb peak in mesohaline and polyhaline conditions differentiated them. In polyhaline conditions, a 25 kb peak was dominant, in contrast to the other conditions where a 35 kb peak dominated (Fig. 2A). The 25 and 60 kb peaks increased seaward.

We observed very few viral clusters shared between salinity niches (Fig. 2D). We found a significant difference in the alpha diversity of viruses between salinity levels (Kruskal–Wallis test for Shannon index, chi-squared = 26.222, *P* value = 8.568e-06 < 0.05). Specifically, both observed and estimated richness and diversity were significantly higher in oligo- and mesohaline samples than in freshwater samples and polyhaline samples (Table S1). Polyhaline samples were underrepresented in our analysis but also exhibited significantly lower diversity and richness than the less saline samples (Fig. 2B). Samples clustered by PCoA based on vOTUs had a stronger differentiation (Fig. 2C) by salinity level than by sampling stations, whereas grouping based on vOTU transcription and expression was comparable, with slightly stronger statistical values for sampling stations (Table 1; Fig. S4A through C). Principal component analyses of whole viromes (Fig. S4D through F) had usually significant, stronger differentiation by salinity level with exception in expression data (Table 1).

**TABLE 1:** Analysis of similarities test results for different data sets

Data set	Salinity level	Sampling station
Metagenome	*R* = 0.4011, *P* = 0.0001	*R* = 0.2632, *P* = 0.0001
Metatranscriptome	*R* = 0.6996, *P* = 0.0001	*R* = 0.3749, *P* = 0.0001
Expression data	*R* = 0.2945, *P* = 0.0001	*R* = 0.3432, *P* = 0.0001
vOTUs	*R* = 0.3843, *P* = 0.0001	*R* = 0.2821, *P* = 0.0001
vOTU activity	*R* = 0.3391, *P* = 0.0002	*R* = 0.3809, *P* = 0.0001
vOTU expression	*R* = 0.3295, *P* = 0.0001	*R* = 0.3648, *P* = 0.0001

**Fig 3:**
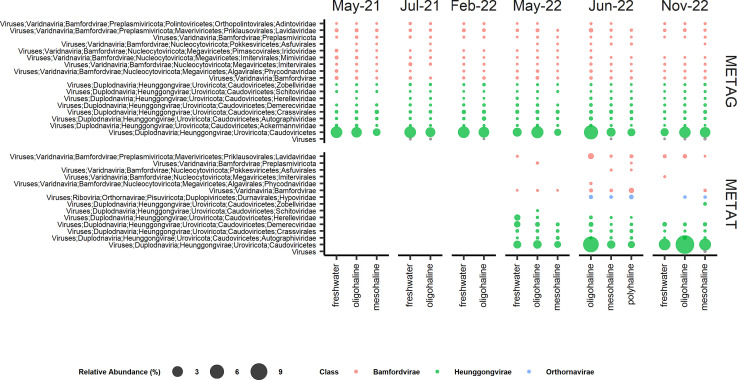
Taxonomic composition of viruses at different salinity levels in metagenomic (METAG) and metatranscriptome (METAT) data in years 2021–2022.

In the estuary, we found a dominance of bacteriophages of the Caudoviricetes. The eukaryotic giant viruses (Megaviricetes) with their satellite viruses (Maverivicetes) were more abundant during the warm seasons (Fig. S5). The less abundant class of viruses was Polintoviricetes, represented by the animal-associated lifestyles, e.g., of the Adintoviridae family (63). A noticeable number of viral contigs could not be taxonomically assigned. We could clearly distinguish taxonomic differences between viruses in different salinities (Fig. 3). Megaviricetes and Maverivicetes occurred in all salinity conditions but were more abundant at lower salinity. The Iridoviridae was the only Megaviricetes family that was not present in polyhaline conditions. Polintoviricetes (dsDNA eukaryotic viruses) occurred predominantly in fresh and brackish waters. Only a single RNA virus family was detected—Hypoviridae—fungal viruses belonging to the class of Duplopiviricetes. Our data showed that most identified viruses were bacteriophages, especially dominating in the brackish and marine conditions.

**Fig 4:**
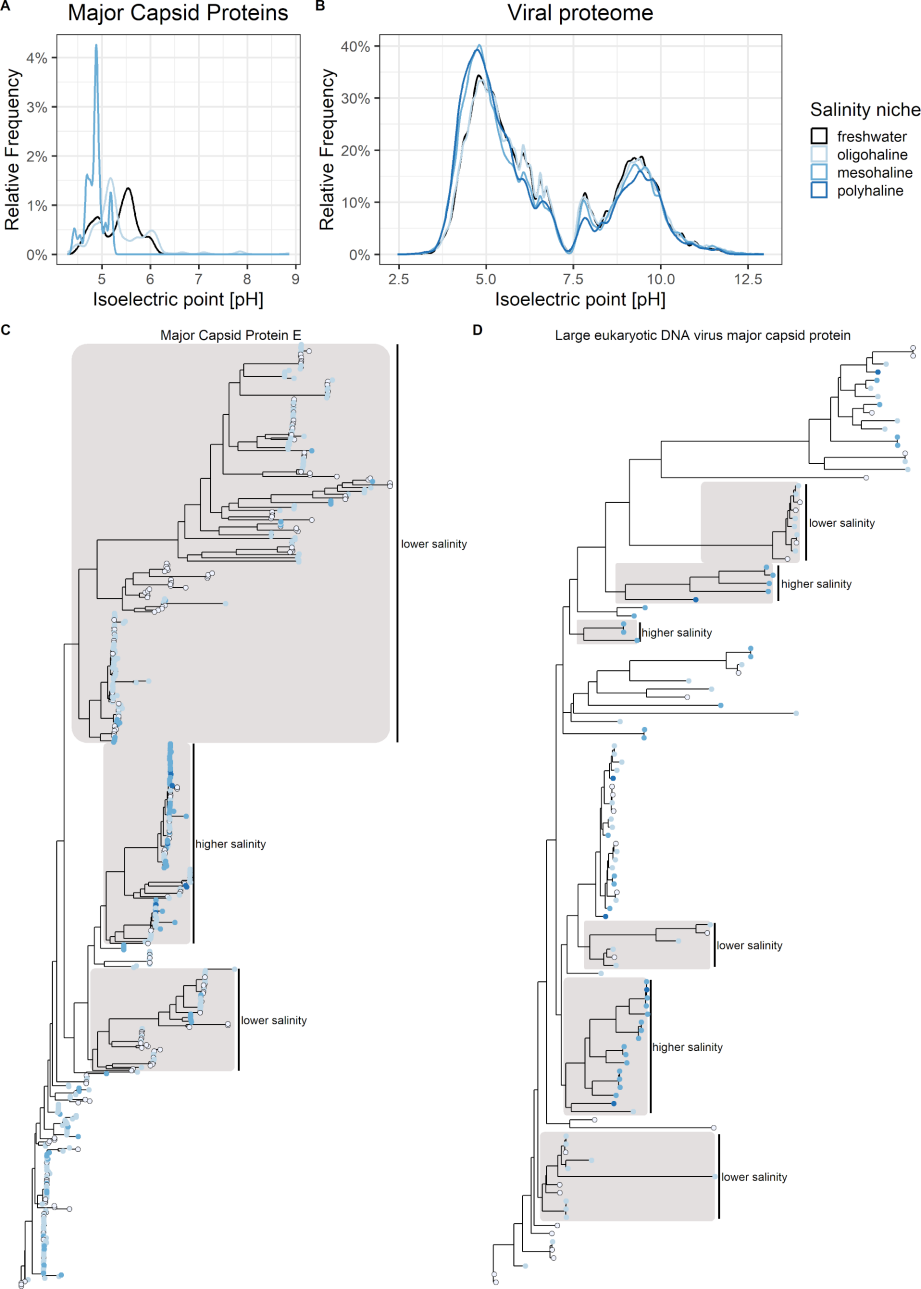
Isoelectric point histograms of (**A**) major capsid proteins (MCPs) and (**B**) predicted viral metaproteome by salinity niche. Too few MCPs were detected in polyhaline conditions for visualization due to their underrepresentation in our data. Phylogenetic analysis of MCPs and color-coded salinity level of corresponding samples for (**C**) MCP E and (**D**) large eukaryotic DNA virus MCP with annotated clusters emerging at lower and higher salinities.

The more resolved phylogenetic analysis of different MCPs, which are commonly used as marker genes for viruses, revealed clades of MCPs originating from the same or neighboring salinity levels for MCP E and large DNA eukaryotic virus MCP (Fig. 4C and D). There were also distinctions in the predicted isoelectric points of viruses metaproteome and MCPs (Fig. 4A and B) from different salinity niches with a gradual shift towards acidity with increasing PSU.

**Fig 5:**
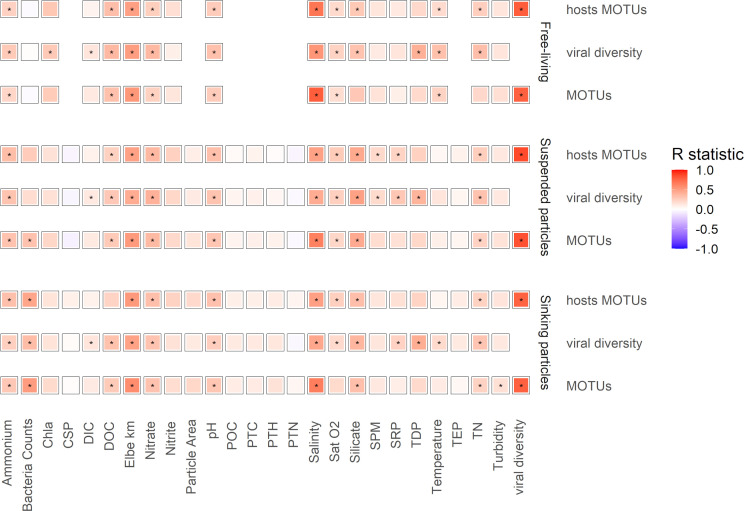
Based on metagenomic data, a pairwise Mantel test between environmental variables and microbial, host, and virus diversity for free-living, suspended, and sinking particle fractions. Color indicates strength and direction of Mantel R statistics, and stars denote adjusted *P* values (*P < 0.05). Abbreviations: Chla, chlorophyll concentration; CSP, Coomassie blue-stained particles; DIC, dissolved inorganic carbon; DOC, dissolved organic carbon; POC, particulate organic carbon; PTC, particulate total carbon; PTH, particulate total hydrogen; PTN, particulate total nitrogen; Sat O_2_, oxygen saturation percent; SPM, all (suspended and sinking) particulate matter per fraction; SRP, soluble reactive phosphate; TDP, total dissolved phosphate; TEP, Alcian blue-stained transparent extracellular particles; TN, total dissolved nitrogen.

Canonical correspondence analysis showed clear ordering of the viral niches along the salinity variable that was significant in most data sets, except for viral activity on sinking particles (Fig. S5). Temperature appeared as significant for free-living and suspended particle-associated viromes in our viral communities and for both particle-associated fractions in the viral activity data. These results were supported by a Mantel test, as salinity is significant for all viral community fractions, and temperature was significantly correlated with viral communities in free-living and sinking particle fractions (Fig. 5). Additionally, total dissolved phosphate significantly influenced viral communities and viral expression in each fraction (Fig. 5; Fig. S7).

### The viruses’ auxiliary genes’ abundance and transcription exhibit spatial patterns

**Fig 6:**
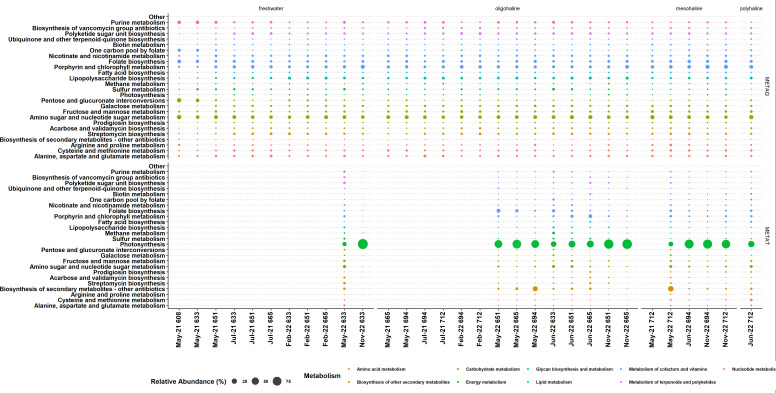
KEGG metabolic pathway categories of identified top viral AMGs constitute 80% of total expression according to their relative abundances at different salinity levels. The upper row represents metagenomic (METAG) data, and the lower row metatranscriptomic (METAT) data.

Metabolic processes and pathways of auxiliary metabolic genes (AMGs) exhibit more spatial than seasonal distribution patterns. Transcriptomic data show variability in pathways reflecting dynamic responses to changing conditions (Fig. 6). We detected pathways related to carbon metabolism, e.g., “photosynthesis” and “methane metabolism.” We analyzed spatiotemporal distributions of two viral auxiliary genes: *psbA* (photosystem II P680 reaction center D1 protein gene) and *alaS* (5-aminolevulinate synthase gene, responsible for a tetrapyrrole precursor of heme, chlorophyll, and vitamin B_12_ synthesis (64) in relation to their prokaryotic equivalent. Generally, the virus to prokaryote ratio of these genes increased seaward, but the opposite tendency was noted for transcriptomic data of *psbA* (Fig. S8 and S9).

**Fig 7:**
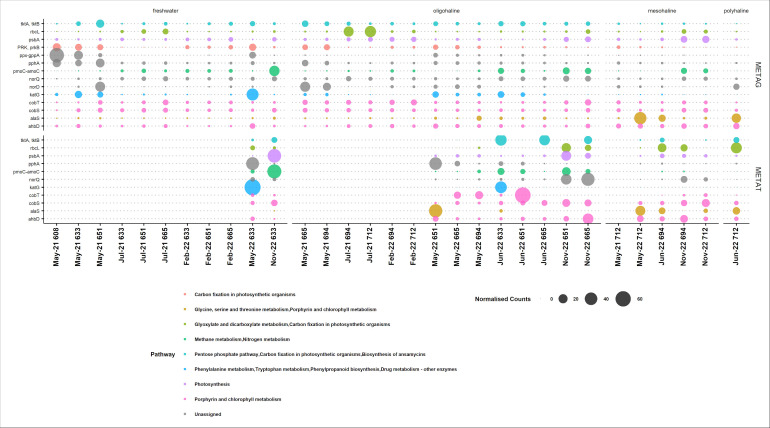
Normalized abundance and transcription counts per auxiliary gene potentially affected by salinity across spatial–temporal scales. PmoC and amoC genes are not distinguished due to their close evolutionary relation.

Among AMGs, we selected those potentially affected by salinity (Fig. 7). Genes related to biogeochemical pathways, such as *norD*, *norQ* (nitric oxide reductases), *amoC* (ammonia monooxygenase subunit), *pmoC* (methane monooxygenase subunit), *ppx-gppA* (exopolyphosphatase/guanosine-5′-triphosphate, 3′-diphosphate pyrophosphatase), *pphA* (serine/threonine protein phosphatase 1), and *tktA*/*tktB* (transketolase), show higher transcript and/or gene abundances in the lower salinity. We observe a similar tendency for *katG*, involved in the oxidative stress response, and for *psbA*, *prkB* (phosphoribulokinase), and *rbcL*, involved in photosynthesis. However, *rbcL* transcription was higher under higher salinity conditions. The genes and transcripts associated with heme biosynthesis, like *ahbD* (AdoMet-dependent heme synthase) and *alaS*, are more abundant in higher salinity. We observe the opposite trend for *cobT* (cobaltochelatase) involved in vitamin B_12_ biosynthesis. *CobS* involved in the same pathway does not exhibit a clear salinity pattern.

### Free-living viruses and those inhabiting suspended and sinking particles share a broad core virome

In contrast to salinity, there were no sharp differences between free-living and particle-associated viruses (Fig. S10). Free-living and particle-associated viromes shared 319 (42.3%) taxa, with 645 (85.5%) shared between the two particle fractions. Only metatranscriptome-derived metabolism profiles revealed subtle differences between the two particle fractions (Fig. S11). There were differences in primary drivers between the free-living and each particle-associated virome. Free-living viromes primarily responded to temperature, silicate, and total dissolved phosphate, whereas particle fractions showed only a few differences in significant relationships, i.e., suspended particulate matter for suspended particle-associated viromes, and temperature and bacterial counts for sinking particle-associated viromes (Fig. 5).

## DISCUSSION

Our work represents one of very few metaomics studies exploring viruses in estuaries on a genetic and transcriptomic level and the first one in the Elbe Estuary. We found a rich diversity of aquatic viruses in the estuary dominated by dsDNA bacteriophages but also including larger viruses likely interacting with eukaryotic phytoplankton. Thereby, a strong differentiation of viral populations based on salinity, without specific adaptations, suggests a strong host dependency of freshwater and marine viruses.

The fact that most of the viruses with predicted hosts have solely one assigned host suggests that most of them are specialists. For some viruses, however, unassigned hosts could be an indication of a broad host range, which seems to be more common than it was previously thought (65). Here, we show the estimated viral genome length distributions in different salinity niches and identify similar trends previously detected by Kavagutti et al. (66), i.e., a close to bimodal distribution of viruses’ genome length and the presence of two dominant peaks at ca. 35–40 kb and 60 kb. The latter increases more towards marine environments. Moreover, we identified a previously unknown peak at 25 kb, which increases with salinity (Fig. 2A). Even though the previously reported peak at 16 kb for freshwater is low in our study, we identified several shorter genomes <20 kb in less saline waters. This suggests that the freshwater environment may be more favorable for smaller viruses. Aside from such differences, the repetitive tendencies between the studies validate our virus detection, while differences in the estimated viral genome length distribution between salinity levels seem to indicate a virus composition specific to salinity niches.

Most of the detected viruses belong to either one or two neighboring salinity niches (Fig. 2D), suggesting unique viral populations. The lack of taxa ranging from freshwater to polyhaline waters indicates the emergence of brackish viral communities, which are not just a simple mixture of freshwater (riverine) and marine (North Sea) communities. The spatial zonation of viruses based on their abundance was previously reported in the Cochin Estuary, India (67), but no sharp distinction was noted on their genetic level (34). While many viral species can be shared between different locations across the estuary (68), it seems that there are vOTU differences, reflected in this study by distinct viral clusters. Indeed, alpha-diversity indexes are mostly significantly different between salinity niches (Table S1; Fig. 2B). Our results show a sharp decrease of alpha diversity in waters with higher salinity, which is consistent with previous studies (68, 69), but it can be biased by the lower number of sampling points in these conditions. The phylogenetic analysis of MCPs reveals multiple clades of proteins emerging from different salinity conditions (Fig. 4C and D) for phage MCP E and for large eukaryotic DNA viruses’ MCPs, indicating separate viruses’ evolution within salinity niches and underscoring salinity as a driver of viral speciation. Moreover, the prediction of the isoelectric point of the viral metaproteome and MCPs (Fig. 4A and B) reveals previously reported shifts towards acidity (70). This notion suggests that viral proteins and especially capsid proteins, which are directly exposed to ionic strength, are well adapted to different osmotic conditions. Salinity drives the occurrence of Megaviricetes and Maverivicetes across time and space in the estuary. Megaviricetes are giant viruses infecting eukaryotic unicellular organisms, while Maveriviricetes are satellite viruses that use the “infecting machinery” of giant viruses for co-infection (71). We found that their co-infections are more likely to occur in summer and localized to freshwater/brackish conditions (Fig. 3). The higher abundance of plankton communities in the station upstream Hamburg Harbor partially explains this tendency (25). Supporting our results, in a previous study exploring multiple metagenomes from diverse biomes, Maverivicetes tended to originate from freshwater samples, and *Caudovirales* were the most ubiquitous viral taxa (12). The lower abundance and activity of giant viruses and their satellite viruses in the brackish waters could be explained by potential co-infection of Polintoviricetes, as according to reports, giant viruses can be suppressed by Polinton-like viruses (72, 73), which are related to Polintoviricetes (74). We detected RNA viruses (*Duplopiviricetes*) only in polyhaline water, and the only detected family—*Hypoviridae*—are fungal viruses. Our data suggest a higher abundance and importance of *Hypoviridae* at the marine (North Sea) sites (Fig. 3). This could be due to the fact that VIBRANT decreased RNA virus recovery in comparison to DNA viruses (39). We acknowledge this as a limitation of our study as we believe that RNA viruses which frequently have eukaryotic hosts play a profound role in ecosystems. Most of the available tools are more suited for DNA viruses leading to biases in RNA virus representation, but recent advancements in RNA virus discovery will increasingly overcome this limitation (75–78).

The clear ordering by viral salinity niches according to the salinity variable in the canonical correspondence analysis plot confirms the correctness of the assigned niches to the viral clusters and highlights salinity as a main grouping factor of different viral populations (Fig. S6). The importance of salinity and temperature for shaping viral communities is supported by a Mantel test and canonical correspondence analyses (28, 68). Viral production has been shown to increase with temperature (29), which relates to increased cell densities, subsequent viral encounter chances, and successful infection rates (28, 29). We previously demonstrated high tryptophan-like DOC (indicating protein origin), together with relatively low particle dry weights during sampling in May and June 2022 (26), which may be related to high viral lytic infection rates during the warm, growing seasons. Infections are more prevalent in polyhaline than in mesohaline waters within estuaries (29). Tidal effects on viral abundance, production, and infection rates were more pronounced upstream and during spring tide, when the associated seawater intrusion resulted in the highest increase in salinity across the macrotidal Jiulong River Estuary (30). Similarly, viral infectivity can be indirectly influenced in the Elbe Estuary by hydrodynamic processes that affect salinity. This notion agrees with increasing viral to prokaryotic ratios of both *psbA* and *alaS* genes along the salinity gradient (Fig. S8 and S9). The viral *alaS* gene, previously reported as prevalent in peat bog habitats (79), may also play a significant role in the Elbe Estuary, which is greatly influenced by terrestrial wash-in of organic matter (to a large extent humic matter) from adjacent marshes (26). Higher temperatures and salinities have a synergistic effect on virus-to-prokaryote ratios, indicating actual infection rates, according to data from the monsoon-influenced estuary (31). Other parameters revealing significance in the Mantel test or CCA analyses, such as oxygen, nitrogen, and phosphate, were previously reported to primarily shape viral communities in estuaries (28, 30, 31, 68).

Salinity can influence viral communities in many ways. First, it is a selection of microbial communities based on their adaptation to osmotic stress. This reduces the effectiveness of viral infection due to an incompatibility of available hosts. Previous transplant studies indeed showed host range differences between marine and estuarine viruses, whereby estuarine viruses seem to be more effective in infecting marine bacteria than vice versa (80). The Mantel test results show that both viral and host communities are correlated with salinity, and hosts are correlated with viruses (Fig. 5), indicating that salinity directly influences hosts’ composition and thus indirectly shapes viral communities. A strong coupling between bacterial and viral abundance was shown before (30, 31, 69). However, the widely distributed broad host range viruses may weaken this relation (81), leading to a discordance between the prokaryotes most affected by salinity and the host of the most affected phage (82). Indeed, in our study, the correlation between viruses and hosts is not perfect, especially in the free-living fraction. This suggests that other factors play an important role in shaping viral communities. Second, it is the direct influence of salinity on viral survival and infectivity. Our isoelectric point analysis suggests that viruses are adapted to different salinity levels, which partially answers the question whether salinity selects for specific viruses based on their osmotic preferences. For example, it has been shown that salt can affect viral capsid stability (83–85). One study showed that an enveloped virus was more susceptible to higher salt concentrations in droplets than a non-enveloped one (86). Furthermore, cross-transplant studies showed that sea and riverine water decreased viral production for non-native viruses, whereby riverine freshwater viruses were more sensitive. Surprisingly, the decay rates for both conditions were reduced in comparison to the respective controls (87). It is possible that salinity has an opposite or concordant influence on both viral and microbial fractions, depending on other local factors (82). Third, it is the physical separation of different communities due to water density differences. Viruses could be more affected as they are less mobile than their hosts. This was concluded before, as phages are more sensitive to dispersal in comparison to prokaryotes due to, e.g., water mixing (82). To verify which role each of the above aspects plays, more research is required, including additional infection experiments on Elbe’s native communities and improved ecological modeling.

We cannot ignore the correlation between salinity and other spatial gradients, which is high but not perfect (Pearson correlation: 0.703, *P* value: <2.2–16 [36]). Although there is definitely a spatial effect on microbial and viral communities influenced, e.g., by urbanization levels and water discharge, it does not explain the sharp viral niche differences we have observed. Principal component analysis shows that data are clustered more by salinity than by sampling station characterized by other local factors, which is confirmed by ANOSIM for metagenomic virome data. The variance explained by salinity is twice the variance explained by station (*R*^2^ = 16% and *R*^2^ = 7%, respectively; Table 1). It also highlights the high variability of salinity at the stations during different time points and seasons, enabling the partial disentanglement of these two factors (Fig. 4). The differences in the ANOSIM results, i.e., in favor of salinity in metagenomic data and sampling station in expression data, can be explained by salinity driving viral communities and spatial factors affecting the actual viral activity.

Because viral sequences were obtained from the microbial-fraction, our virome is enriched in a more active fraction of viruses. These are mainly viruses attached to the cell, actively encountering/lysing cell viruses and integrated into a genome as proviruses when most of the free-living viruses passed through a 0.22 µm filter (88). Therefore, we used expression as an appropriate approach to select the most important pathways viruses were involved in. Many of the detected viral pathways can be associated with host infection; e.g., the “lipopolysaccharide biosynthesis” pathway is potentially involved in virus entry into and release from the host cell, whereas the photosynthesis pathways are known to be manipulated by cyanophages, enabling energy transfer to viral protein translation (89). In the metatranscriptomic data, we detected viral genes (e.g., *pmoC*) known to alternate host methane metabolism (90), suggesting an important yet little-studied role of viruses for carbon turnover in the Elbe Estuary. The AMGs related to both methane (e.g., *pmoC*) and nitrogen metabolism (e.g., *amoC*, *norQ*, and *norD*; Fig. 6 and 7) are localized in less saline conditions following a similar pattern to prokaryotic activity derived from the same data set (36). In riverine and estuarine environments, both of these processes are suppressed by increasing salinity (91–93), due to the adaptation of methanotrophs, methanogens, and nitrifiers to specific salinity conditions (94–96). In the Elbe Estuary, high concentrations of inorganic nitrogen were indeed noted close to the Hamburg Harbor, characterized by lower salinity but also by higher human activity. Therefore, other than salinity, the runoff from agriculture, wastewater plants, and general industrialization could play a role here (97). Another important factor could be plankton die-off due to entering light-limited dredged areas caused by a dramatic increase in depth from 5 to 15 m (98). The high concentrations of nitrogen sources are favorable conditions for denitrifiers, which become available hosts for viruses. Indeed, the NorD and NorQ take part in denitrification, and AmoC in nitrification. Viral *norQ* was found integrated into the genomes of the prokaryotes involved in N cycling: Nitrososphaerales and Rhizobiales, and phages assigned to Bradyrhizobium, whose members have been reported to exhibit denitrification activity (99, 100) (Table S2). Similarly, AMGs involved in phosphorus metabolism could support hosts in the effective utilization of the high concentration of phosphates in the upper estuary (26). Surprisingly, *katG*, which regulates oxidative stress, has the highest abundance at lower salinities associated with the upper parts of the estuary, suggesting that it counteracts stresses associated with anthropogenic inputs rather than osmotic stress (101). On the other hand, there are genes more abundant in saline conditions: *ahbD* can function in counteracting osmotic stress in cyanobacteria (102, 103) and extreme halophiles (104); *alaS* has confirmed a role in osmotic stress regulation in higher plants (101); and genes involved in carbon fixation, like *rbcL*, were previously reported as more prevalent in stress environments (105). The AMGs involved in the abovementioned pathways could improve host fitness according to the piggyback-the-winner model (106). Our data (Table S2) agree with the widespread occurrence of the *cobS* and *cobT* genes in tailed viruses, including cyanophages (107, 108). The presence of *cobS* inphages infecting the Roseobacter CHUG lineage aligns with our *cobS* detection in phages assigned to the host belonging to the same family (Rhodobacteraceae) (109). The viral genes encoding cobaltochelatase subunits CobS and CobT could hijack the host’s cobaltamine metabolism by complementing the already present CobN subunit to support viral genetic material replication (110). The higher abundance of these genes could be more related to higher primary production driving the cobalamin demand in the upper part of the estuary than salinity effects (111–113). Apart from that, we noticed pathways related to host defense, e.g., NAD^+^ (belonging to the nicotinate and nicotinamide metabolism pathway), are involved in bacterial immunity and phage counterdefense (114). We detected many genes related to antibiotics and xenobiotic (artificial compounds) metabolism. Although antibiotic resistance genes (ARGs) are not as widespread in bacteriophages as it is commonly believed, they can be present in phages or, more often, in phage–plasmids, which both can be vectors of ARG transmission in the environment (115). The urbanized and agricultural surroundings, high boat transportation traffic in the Elbe Estuary, and other anthropogenic factors are favorable conditions for increasing plasmidome diversity (including viruses in plasmid-like forms) and spreading ARGs (116). Furthermore, the anthropogenic pressure could potentially affect virus–host adaptation, raising the question of the exact role of these AMGs for viral infections. Unfortunately, due to limitations in AMGs annotations, the interpretation of bacteriophage auxiliary genes and pathways remains limited. Thus, special care should be taken, as the function of many genes annotated with pathways related to a broad spectrum of substrates, sulfur, cysteine, and methionine, and folate metabolism likely belongs to essential viral metabolism (117). Yet, the general overview of viral genes may provide hints on their potential role in infection and evolutionary success.

Our results suggest big overlaps of viromes from the different fractions. Especially, many vOTUs were common for both particulate fractions, for which differences are not reflected on the taxonomic level (Fig. S12). However, we can highlight differences between fractions in viral metabolism (Fig. S11). This suggests different viral infection strategies rather than differences in viral communities inhabiting free-living and different particulate fractions.

Here, we provide detailed genetic evidence for distinct viral populations in the Elbe Estuary in relation to known biological and physical processes. We identified possible factors shaping viral populations of the Elbe Estuary with a focus on salinity, which we showed is a primary viral speciation factor. Furthermore, we presented a comprehensive overview of the viral communities in the Elbe Estuary on spatial and temporal scales. Our results can help to link freshwater and marine viral studies and predict future effects of freshwater salinization, e.g., due to increasing sea level (floods), increased road and mining salts, or decreased riverine water levels (droughts).

### Conclusion

Our study highlights distinct viral communities in different estuarine salinity niches with distinct brackish viral populations, independent of marine and riverine viromes. In the presented work, we support previous results that identify salinity as the primary driving factor shaping viral communities. We propose salinity as a speciation factor of viruses in the Elbe Estuary, which selects host communities and viruses based on their adaptation to osmotic pressure and spatially isolates communities by separating water masses due to their density differences. Additionally, we identified temperature and total dissolved phosphate as important drivers of viral communities, as well as their prokaryotic and eukaryotic hosts. We showed that even though free-living and particle-associated fractions exhibit differences on the metabolic level, they share a core virome and that there are specific differences in viral activity between suspended and sinking particle fractions. Our study provides insights into salinity effects and anthropogenic pressure on virus–host relationships through the lenses of virus involvement in metabolic pathways across the Elbe Estuary. It can contribute to further investigations on viral communities, including direct salinity effects on virions and related carbon dynamics.

## Data Availability

Raw sequence reads and assemblies used in this study are available for download via the ENA (PRJEB54081). The R scripts used for data analysis are deposited in the GitHub repository (https://github.com/JuliaGol/Elbe_viruses).

## SUPPLEMENTAL MATERIAL

Supplemental material aem.00807-26-s0001.docx Table S1; Fig. S1 to S12.

Table S2 aem.00807-26-s0002.xlsx AMG orthologs and taxonomic assignment.

ASM does not own the copyrights to Supplemental Material that may be linked to, or accessed through, an article. The authors have granted ASM a non-exclusive, world-wide license to publish the Supplemental Material files. Please contact the corresponding author directly for reuse.
