# Supplementary material for: Salinity-driven niche partitioning of aquatic viruses in one of Europe’s largest estuaries
Source: Appl Environ Microbiol. 2026 Jun 10;92(7):e00807-26. doi: 10.1128/aem.00807-26 (PMC13390420; doi:10.1128/aem.00807-26)
Supplement: Supplemental material — Table S1; Fig. S1 to S12. [file aem.00807-26-s0001.docx]

Supplement

TABLE S1 Dunn test pairwise results for Shannon alpha diversity index

| Comparison | Z | P.unadj | P.adj |
| --- | --- | --- | --- |
| freshwater- mesohaline | -3.12499 | 1.78E-03 | 5.33E-03 |
| freshwater - oligohaline | -4.49051 | 7.11E-06 | 4.26E-05 |
| mesohaline - oligohaline | -0.31281 | 7.54E-01 | 7.54E-01 |
| freshwater - polyhaline | 1.098652 | 2.72E-01 | 3.26E-01 |
| mesohaline - polyhaline | 2.35553 | 1.85E-02 | 2.77E-02 |
| oligohaline - polyhaline | 2.575638 | 1.00E-02 | 2.00E-02 |

[added as supplementary Excel file]

Table S2: List of AMGs orthologs and taxonomic assignment.


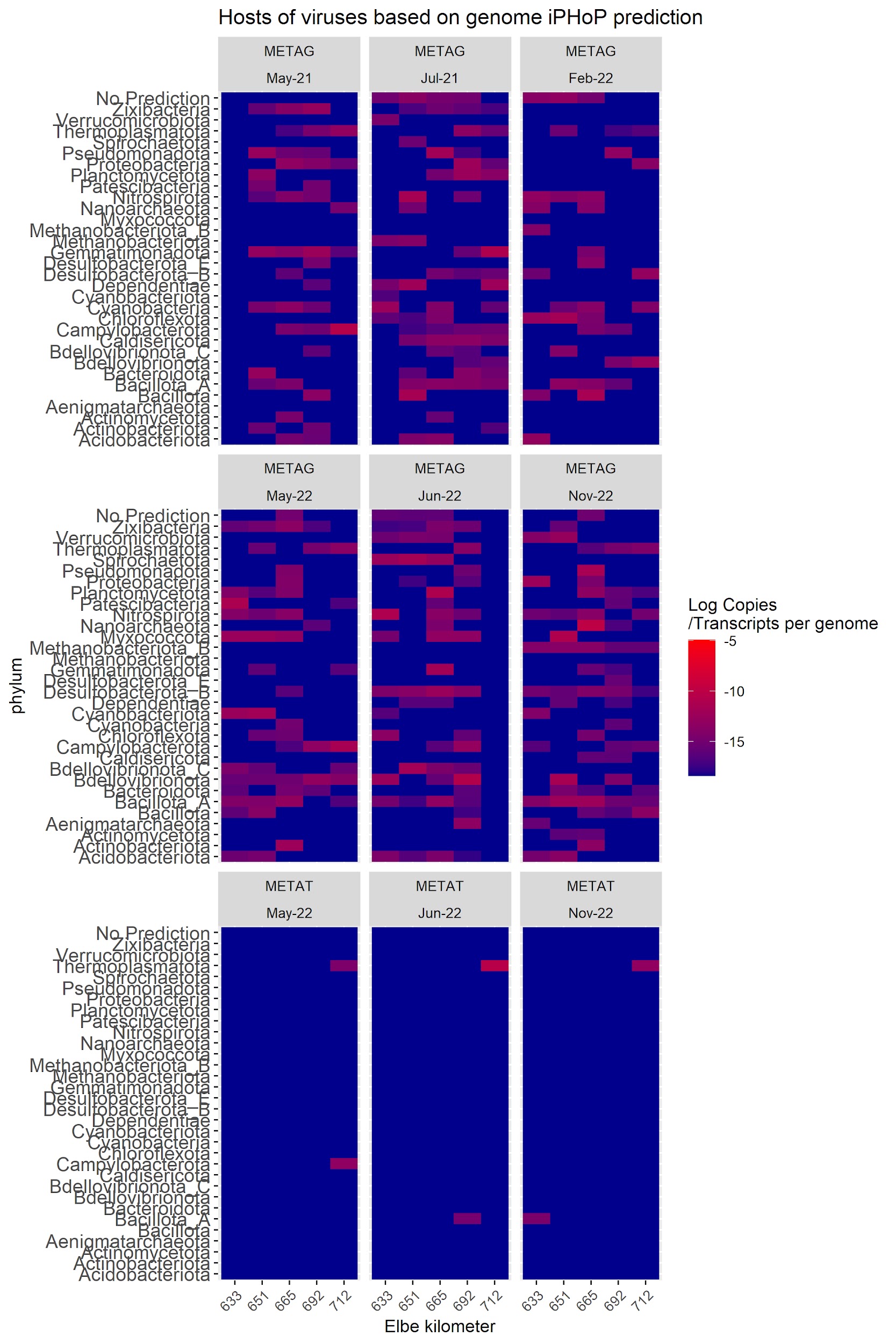


FIG S1 Species level host prediction by iPHoP.

FIG S2 Genus level host prediction by iPHoP.
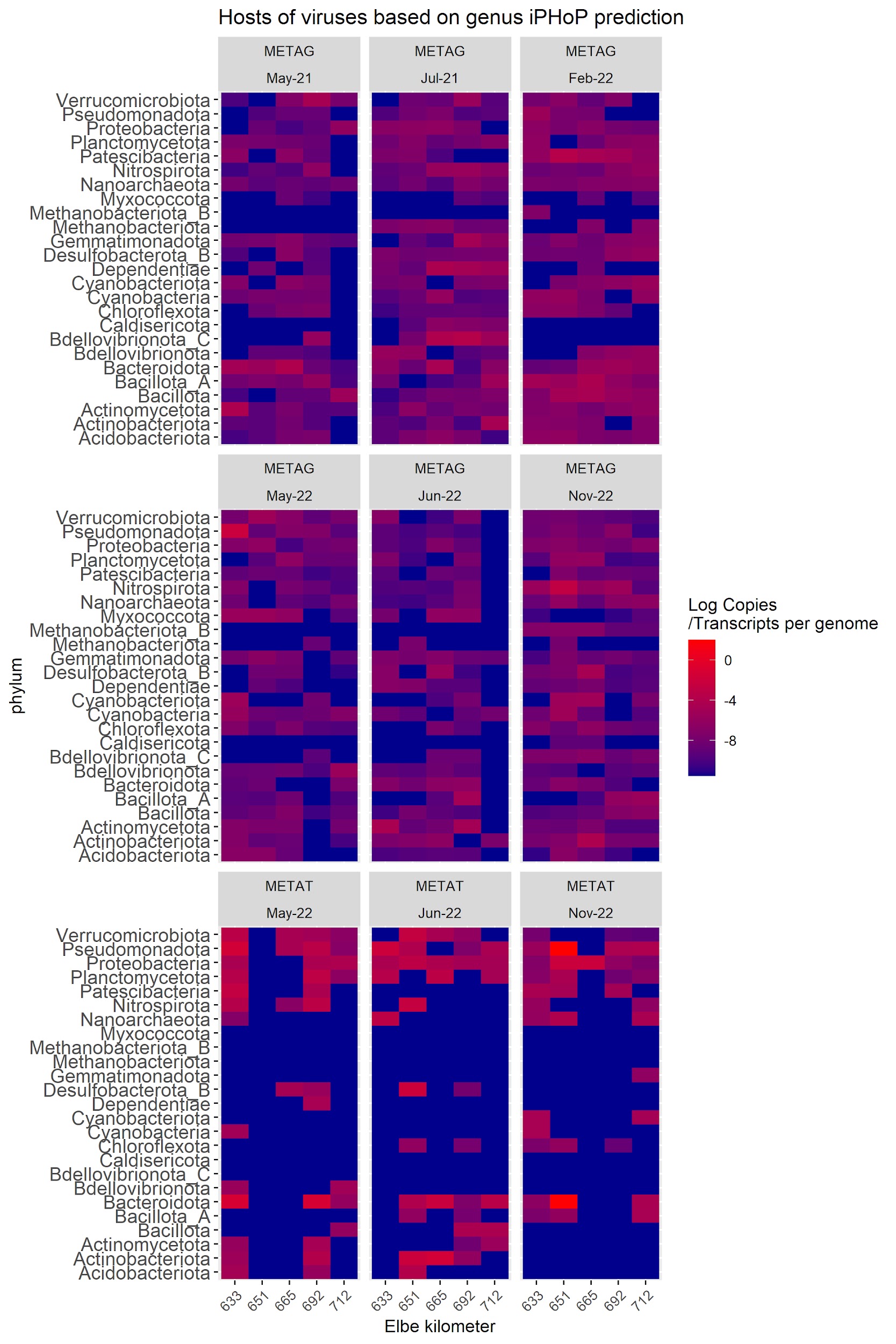


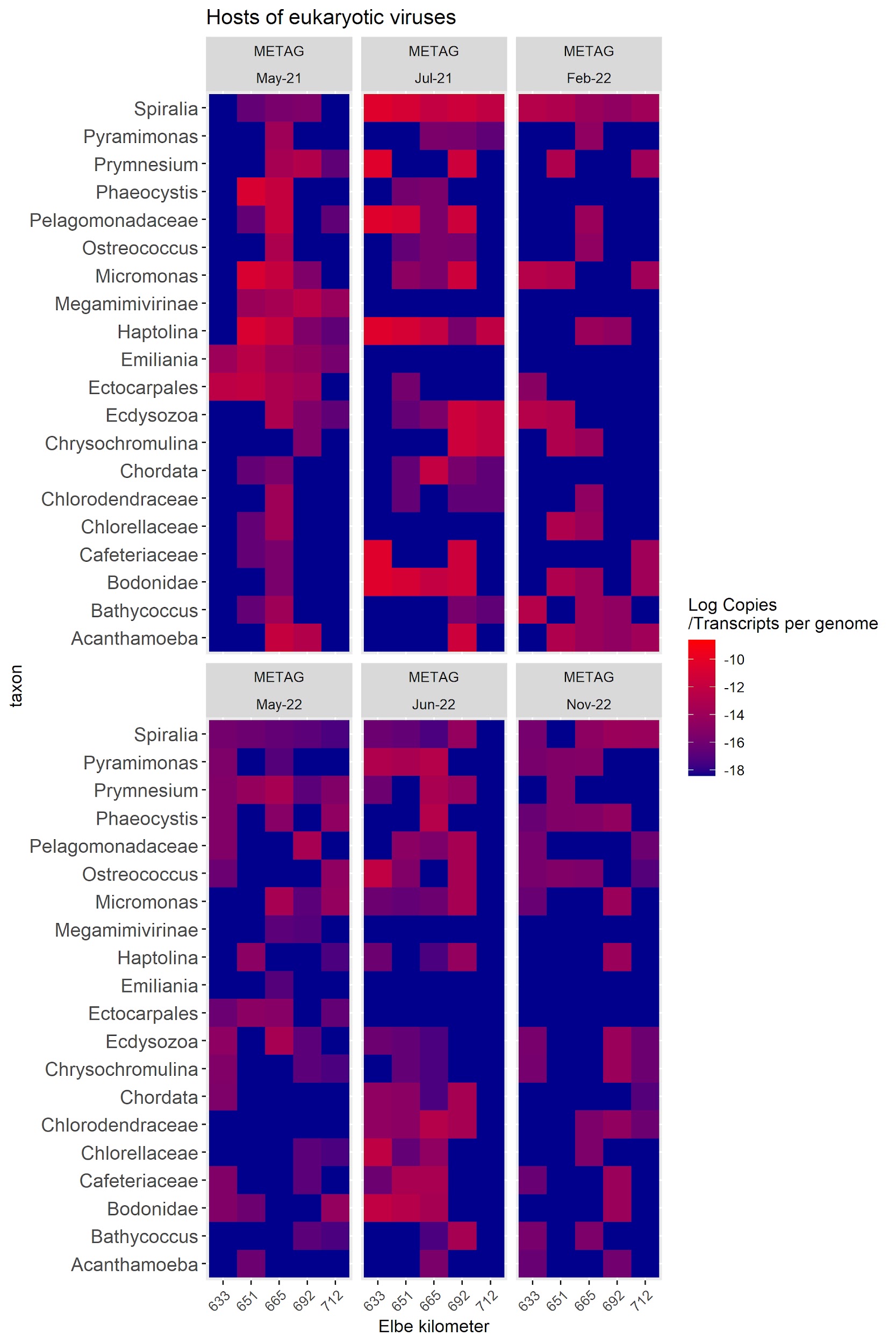


FIG S3 Eukaryotic hosts prediction based on Virus-Host DB.


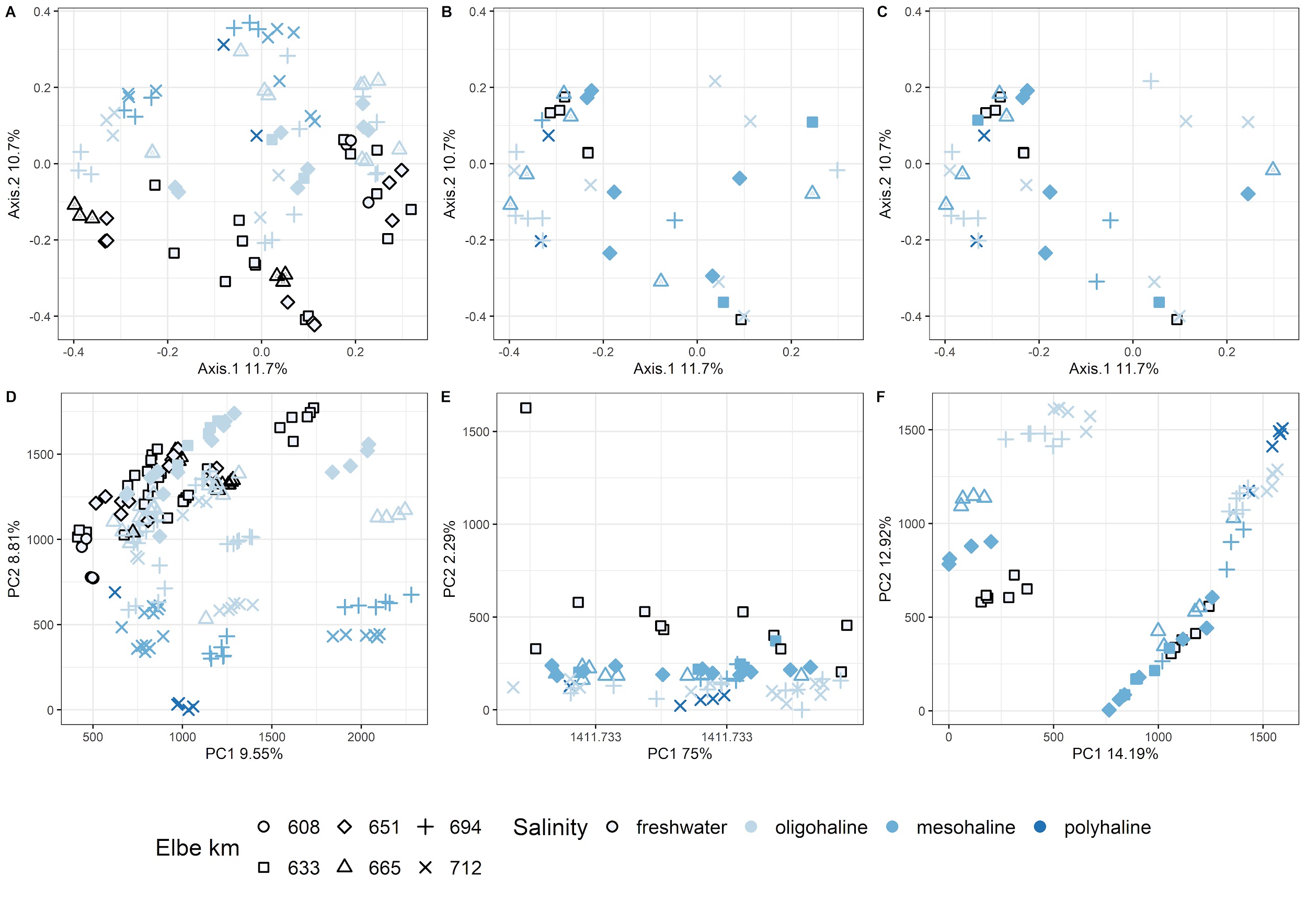


FIG S4 Dimension reduction of datasets used in this publication. PCoA reduction of A) vOTUS, B) transcriptional activity of vOTUs C) expression of vOTUS and PCA of D) virome metagenome E) virome metatranscriptome F) virome expression.


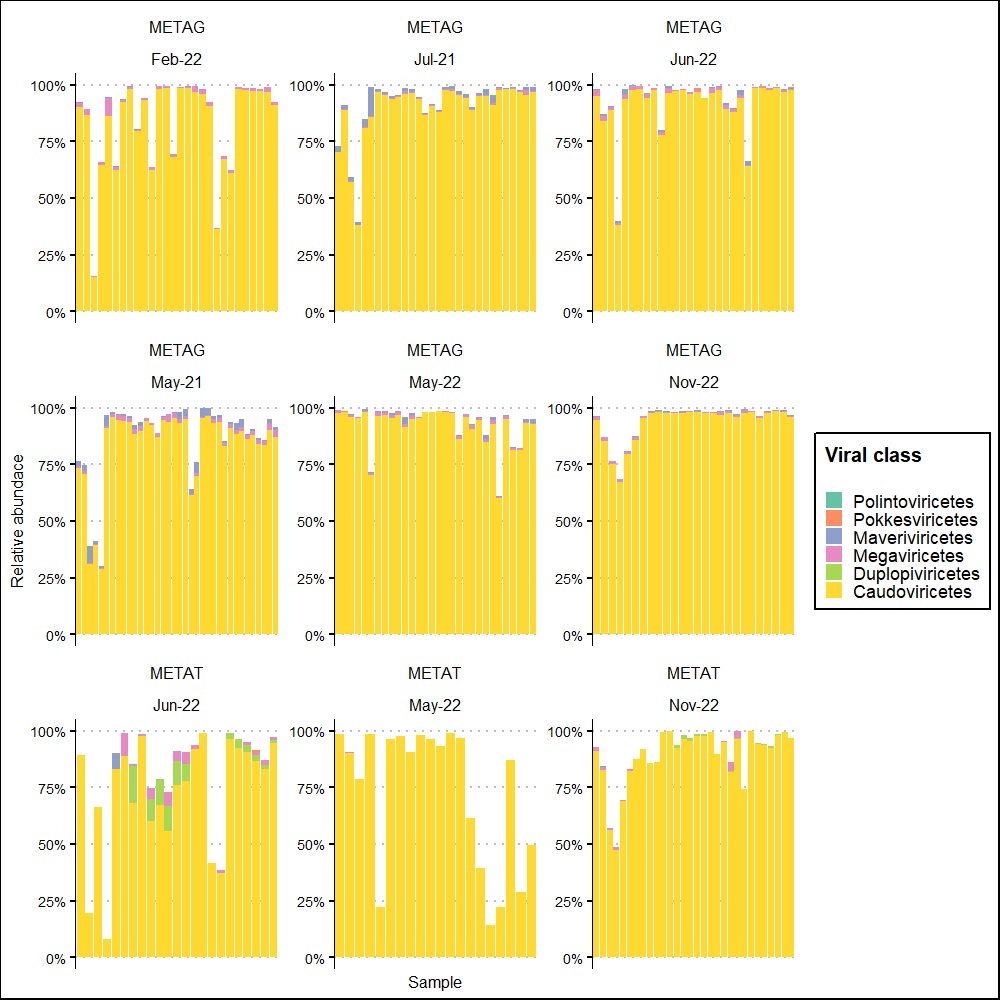


FIG S5 Viral taxonomic distribution according to stations and seasons.


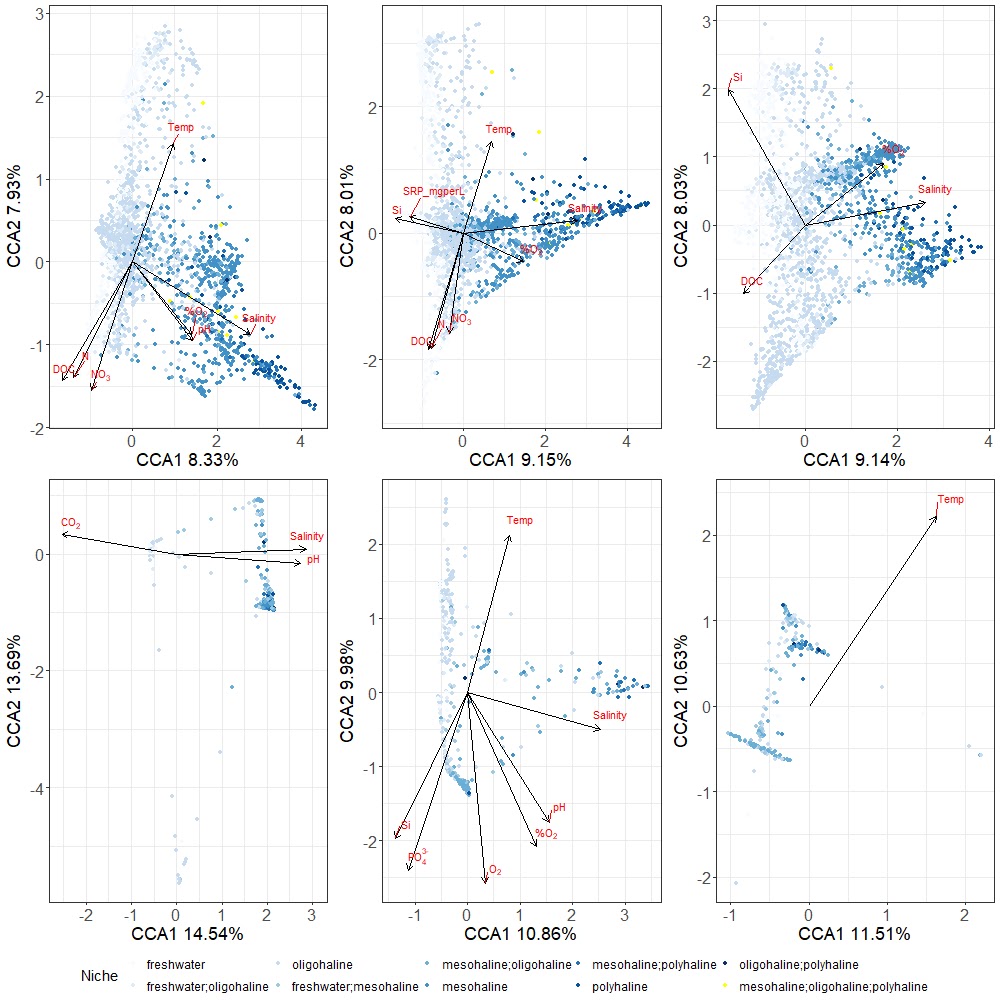


FIG S6 Canonical correspondence analyses from left to right free-living, suspended and sinking particle fractions; In first row - vOTUs from metagenomic data (diversity); in the second row - vOTUs from metatranscriptomic data (activity). Only the significant variables are displayed based on empirical p-values (≥0.001).


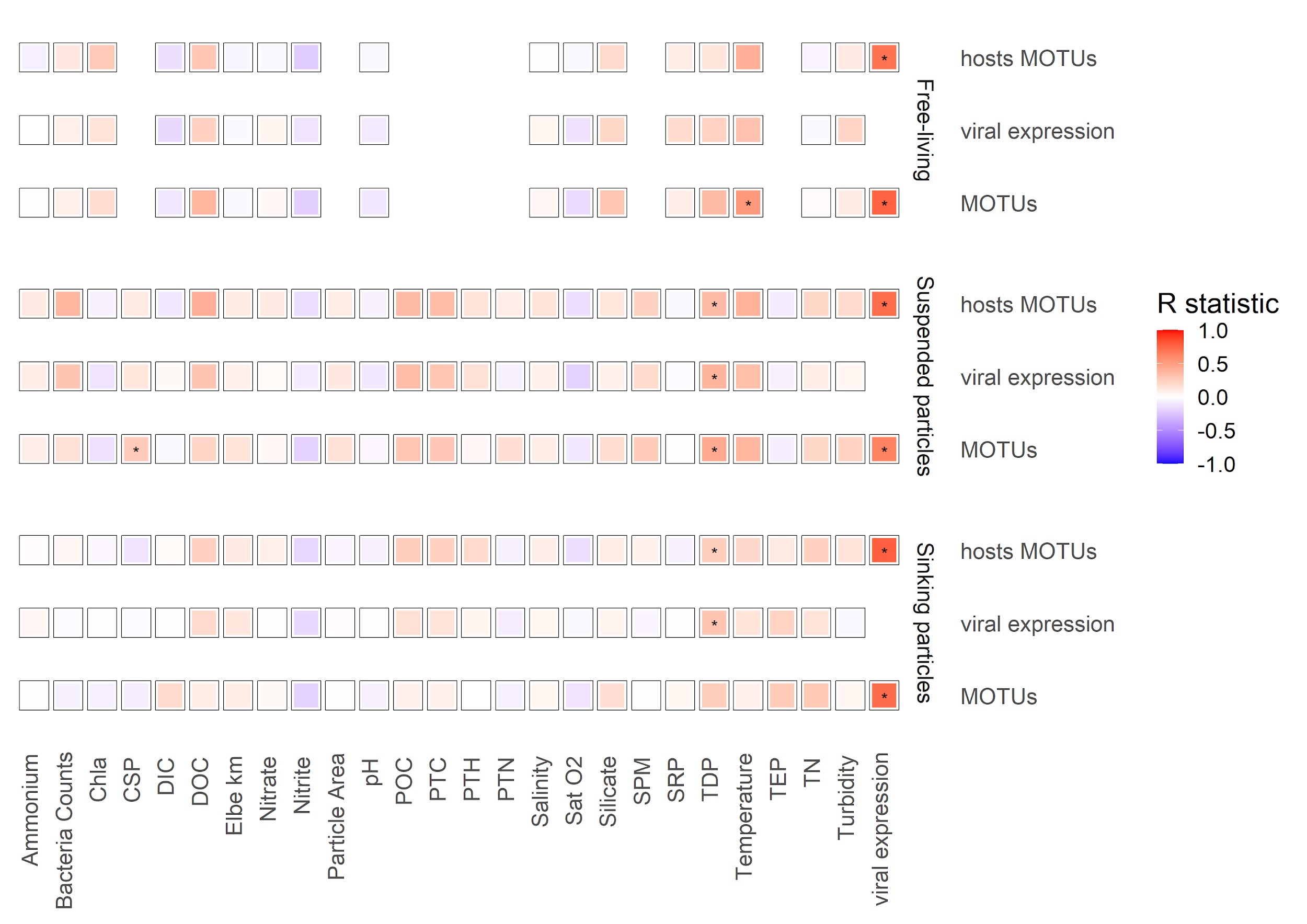


FIG S7 Based on vOTUs expression data pairwise Mantel test between environmental variables and microbial, hosts and viruses expression: for A) Free-living B) suspended fraction C) sinking fraction. Colour indicates strength and direction of Mantel R statistics and stars denote adjusted p-value (*P < 0.05). Shortcuts: Chla - chlorophyll concentration, CSP - coomassie blue stained particles, DIC - dissolved inorganic carbon, DOC - dissolved organic carbon, POC - particulate organic carbon, PTC - particulate total carbon, PTH - particulate total hydrogen, PTN - particulate total nitrogen, Sat O2 - oxygen saturation percent, SPM - all (suspended and sinking) particulate matter per fraction, SRP- soluble reactive phosphate, TDP - total dissolved phosphate, TEP - alcian blue stained transparent extracellular particles, TN - Total dissolved nitrogen.


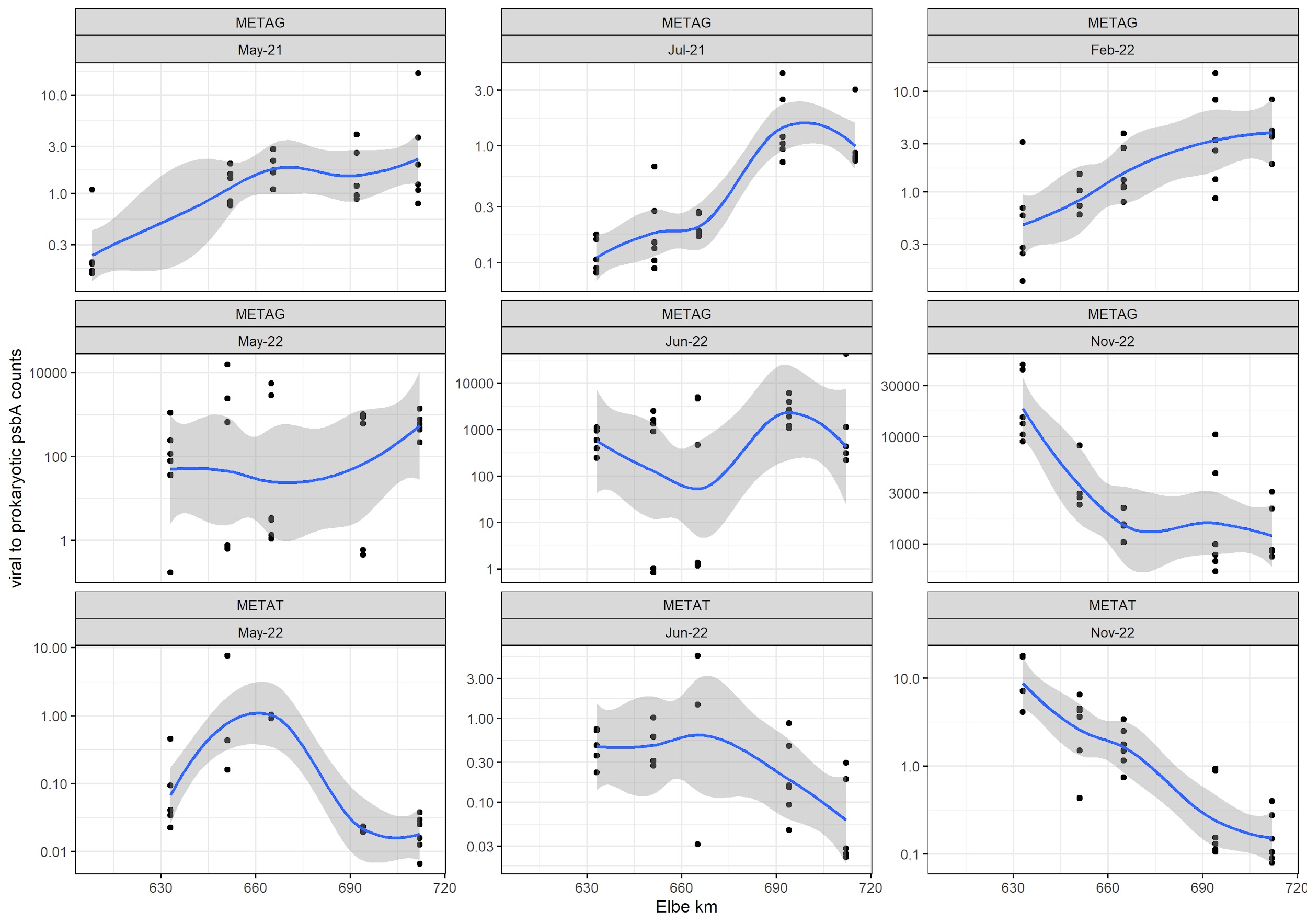


FIG S8 PsbA viral to host counts ratios across spatio-temporal scales.


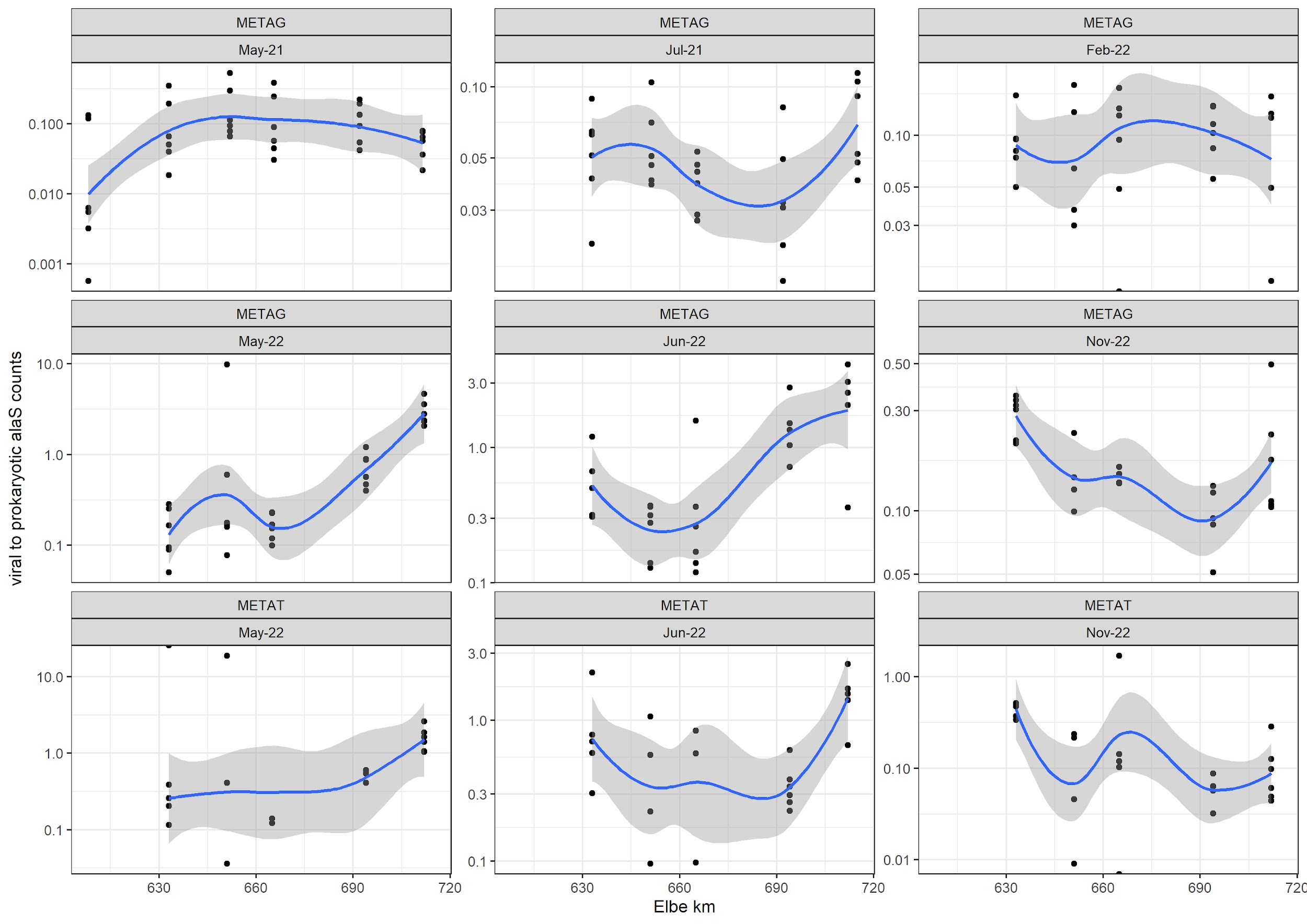


FIG S9 AlaS viral to host counts ratios across spatio-temporal scales.


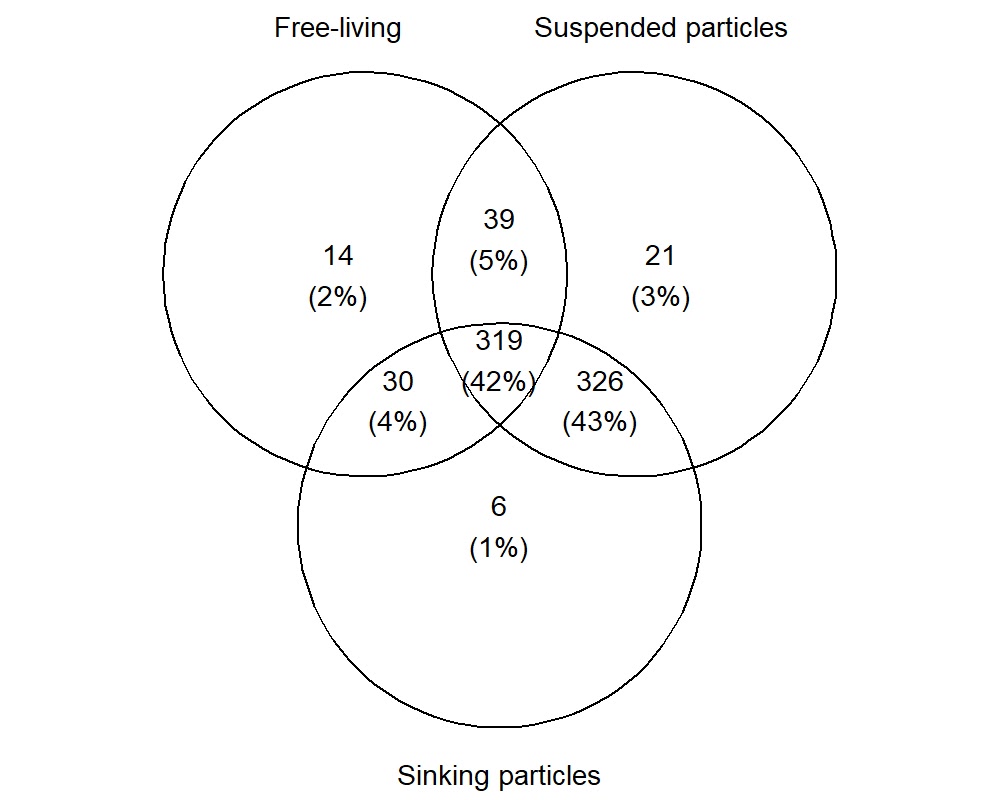


FIG S10 Venn diagram representing number and percentages of viruses in different fraction niches.


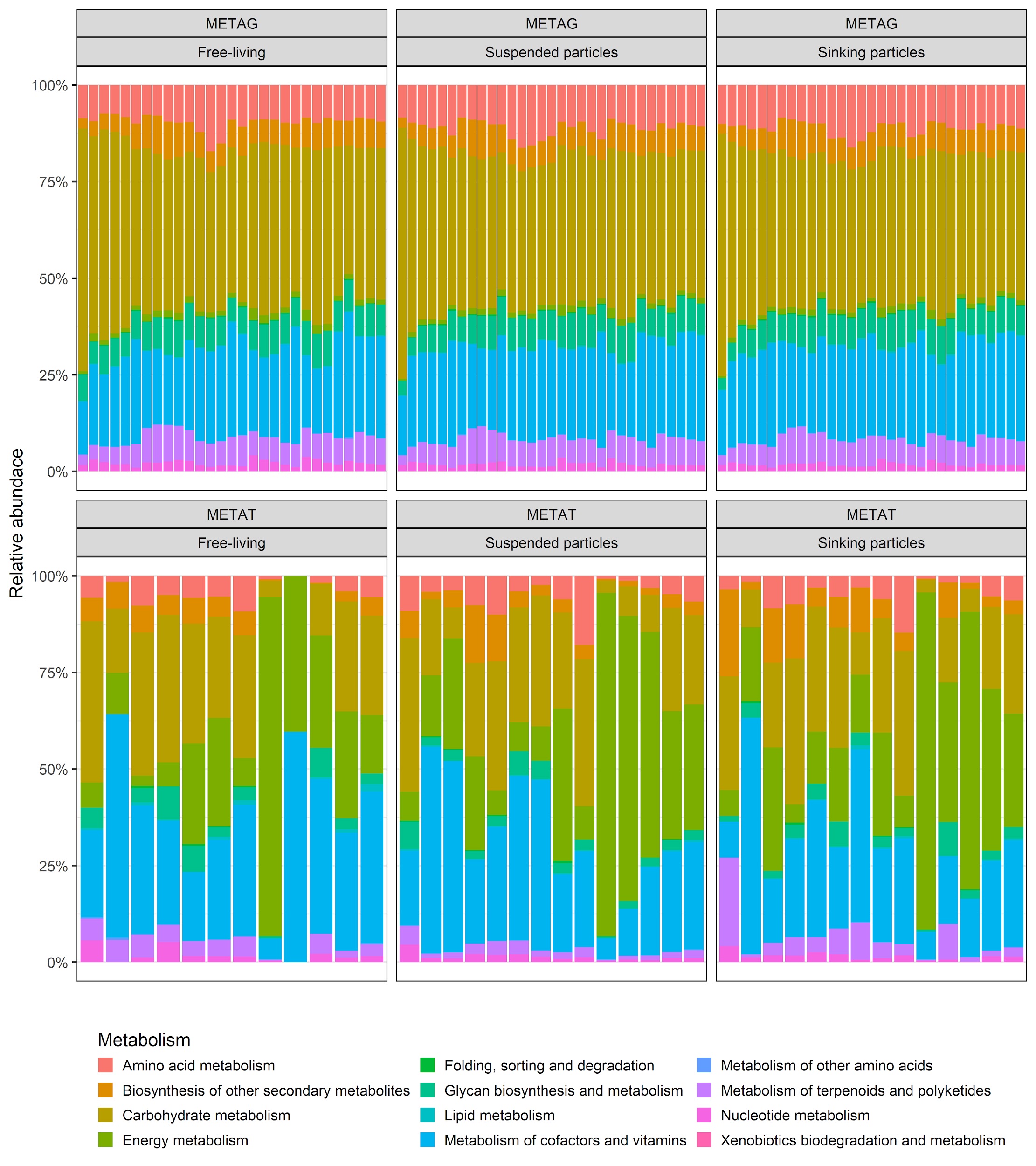


FIG S11 Relative abundance of auxiliary genes grouped by GO metabolism categories according to filtered fraction and data type.


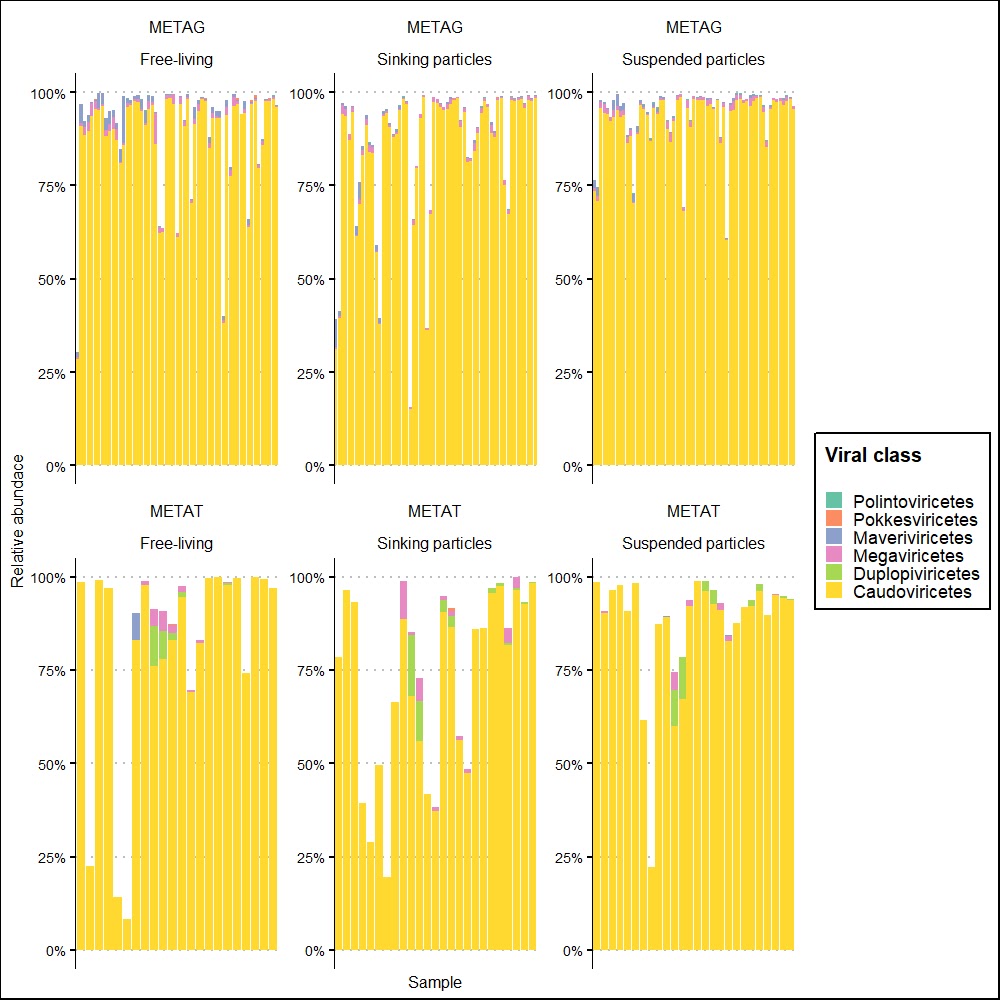


FIG S12 Viral taxonomic distribution according to fractions.
